# Co-Expression of TIGIT and Helios Marks Immunosenescent CD8^+^ T Cells During Aging

**DOI:** 10.3389/fimmu.2022.833531

**Published:** 2022-05-16

**Authors:** Daan K. J. Pieren, Noortje A. M. Smits, Rimke J. Postel, Vinitha Kandiah, Jelle de Wit, Josine van Beek, Debbie van Baarle, Teun Guichelaar

**Affiliations:** ^1^ Centre for Infectious Disease Control, National Institute for Public Health and The Environment, Bilthoven, Netherlands; ^2^ Center for Translational Immunology, University Medical Center Utrecht, Utrecht University, Utrecht, Netherlands

**Keywords:** CD8+ T cells, aging, TIGIT, Helios, influenza, immunosenescence, CD28, CD27

## Abstract

Aging leads to alterations in the immune system that result in ineffective responsiveness against pathogens. Features of this process, collectively known as immunosenescence, accumulate in CD8^+^ T cells with age and have been ascribed to differentiation of these cells during the course of life. Here we aimed to identify novel markers in CD8^+^ T cells associated with immunosenescence. Furthermore, we assessed how these markers relate to the aging-related accumulation of highly differentiated CD27^-^CD28^-^ cells. We found that co-expression of the transcription factor Helios and the aging-related marker TIGIT identifies CD8^+^ T cells that fail to proliferate and show impaired induction of activation markers CD69 and CD25 in response to stimulation *in vitro*. Despite this, in blood of older adults we found TIGIT^+^Helios^+^ T cells to become highly activated during an influenza-A virus infection, but these higher frequencies of activated TIGIT^+^Helios^+^ T cells associate with longer duration of coughing. Moreover, in healthy individuals, we found that TIGIT^+^Helios^+^ CD8^+^ T cells accumulate with age in the highly differentiated CD27^-^CD28^-^ population. Interestingly, TIGIT^+^Helios^+^ CD8^+^ T cells also accumulate with age among the less differentiated CD27^+^CD28^-^ T cells before their transit into the highly differentiated CD27^-^CD28^-^ stage. This finding suggests that T cells with immunosenescent features become prominent at old age also within the earlier differentiation states of these cells. Our findings show that co-expression of TIGIT and Helios refines the definition of immunosenescent CD8^+^ T cells and challenge the current dogma of late differentiation stage as proxy for T-cell immunosenescence.

**Graphical Abstract:**
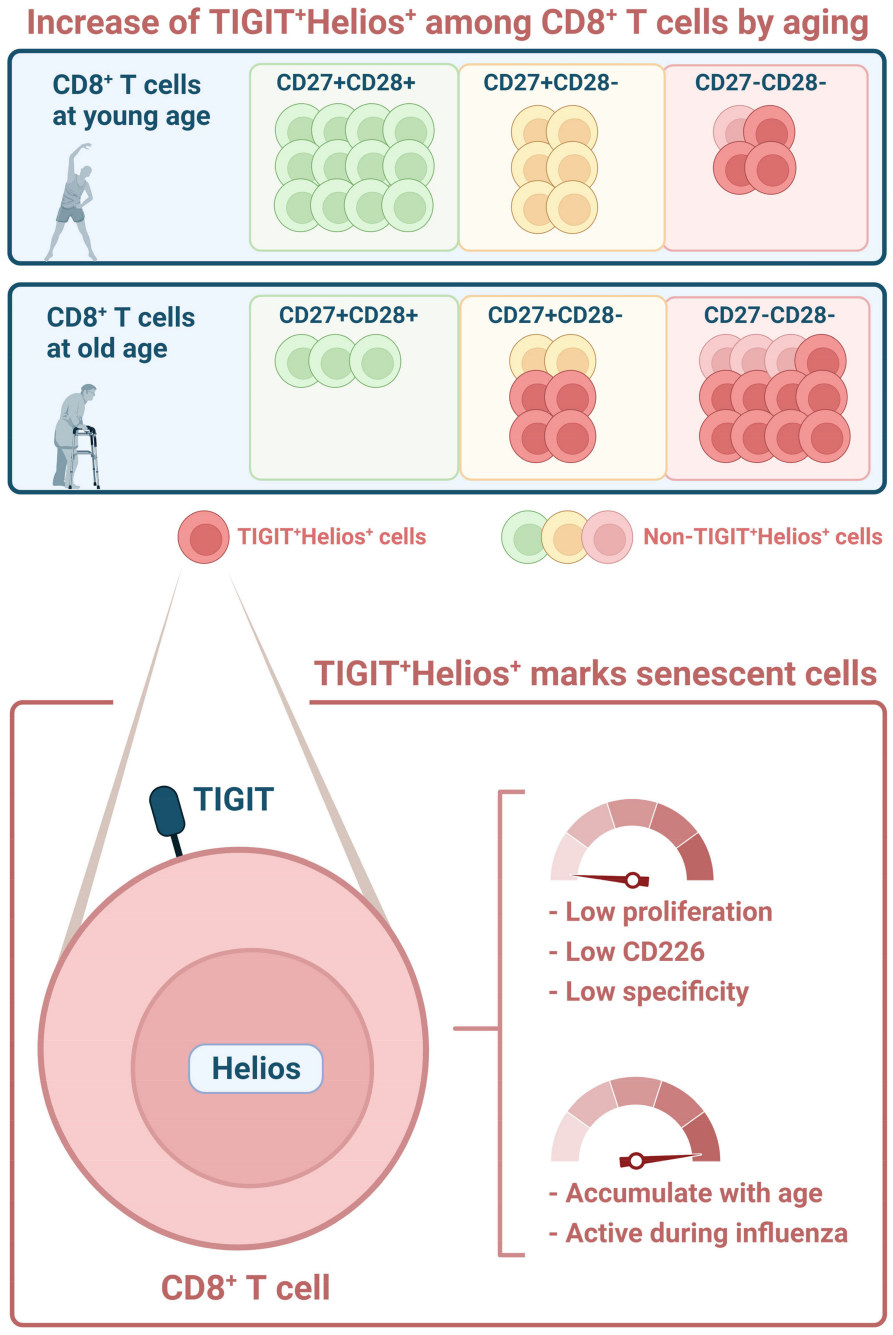


## Introduction

Viral respiratory diseases such as influenza and COVID-19 severely strike the elderly due to changes that aging imposes on the immune system (1–3). Immunological dysfunctions that evolve during aging occur throughout the immune system and are collectively defined as immunosenescence (3). This has widely been reported for CD8^+^ T cells that are required to control viral infections (4) and malignant cells (5). The CD8^+^ T-cell population loses proper responsiveness at old age, as reflected by reduced capacity to become activated and proliferate in response to stimulus (6–9). Immunosenescence can be viewed from many different perspectives, including the expression of immune markers. Hence, it is difficult to achieve a clear view of how T cells change during aging.

Differentiation of CD8^+^ T cells during the course of life leads to accumulation of highly differentiated T cells at old age and gain of features that are associated with immunosenescence (3, 10–12). Progression towards senescence in CD8^+^ T cells has been stratified by three successive differentiation states based on expression of CD27 and CD28 (10, 13). This approach describes a gradual path from the early differentiation state (CD27^+^CD28^+^) *via* an intermediate differentiation state (CD27^+^CD28^-^) into the late differentiation state (CD27^-^CD28^-^) that expresses many features of immunosenescence, such as reduced capacity to proliferate (10). Due to these features, the accumulation of CD27^-^CD28^-^ cells is often used as a proxy for the level of immunosenescence in the CD8^+^ T-cell pool (10, 11, 13, 14).

Although accumulation of late-differentiated T cells may be useful as surrogate marker of immunosenescence progression, it does not fully relate to functional changes in the CD8^+^ T-cell population with aging, such as reduced proliferation and activation. To better understand progression of immunosenescence, it would be helpful to define markers that more precisely pinpoint functionally immunosenescent cells. The co-inhibitory receptor TIGIT has recently been identified as a potent marker of immunosenescence as it is expressed by dysfunctional T cells that accumulate at older age (15). Its clear functional role was shown by blocking TIGIT, which resulted in improved functionality of immunosenescent T cells (15–17). The transcription factor Helios is a marker that may further define different functional subsets among TIGIT^+^ T cells. For example, Helios co-expression with TIGIT has been described in CD4^+^ T cells to define a functionally distinct cell subset that is characterized by regulatory properties (18, 19). Additionally, a significant proportion of CD8^+^ T cells also expresses Helios. Recent studies have suggested an association of Helios with exhaustion in CD8^+^ T cells (20) and an increase of Helios expression with aging among the CD8^+^ T-cell population (21). However, the value of Helios as marker of CD8^+^ T-cell immunosenescence remains to be defined. Here, we explored the expression of Helios combined with TIGIT as marker to better identify CD8^+^ T-cell immunosenescence and how these markers relate to CD8^+^ T-cell differentiation status during aging.

## Materials and Methods

### Study Design

Blood obtained from asymptomatic individuals (n=50, 21-82 years of age) was derived from three different sources: blood donors (Sanquin Blood Supply Foundation), participants of the NVI-255 study at the RIVM (Netherlands Trial Register NTR2070) (22), or community-dwelling older adults from the ILI-3 study at the RIVM (Netherlands Trial Register NTR4818) (23, 24). Blood obtained from symptomatic influenza-A infected individuals was derived from older adults (63-83 years of age, n=15; H3N2 n=12, H1N1 n=3) participating in the aforementioned ILI-3 study (NTR4818), which was performed according to Good Clinical Practice, the Declaration of Helsinki. The study was approved by the ethical committee METC Noord-Holland and written informed consent was obtained from all participants. There were no exclusion criteria for this study. Participants were 60 years and older, and were instructed to report ILI-associated symptoms according to the Dutch Pel criteria (25) (fever ≥ 37.8°C with at least coughing, myalgia, nasal congestion, sore throat, difficulty breathing, or headache) as soon as possible after the symptoms started. Blood samples were drawn within 72 hours after the symptom report (acute phase of infection), and at approximately two and eight weeks after the initial report. The selection of influenza patients from the ILI cohort was based on the confirmed presence of influenza A virus as a single infection at the acute phase of infection and absence of influenza A virus at two and eight weeks after the initial report. The presence or absence of ILI-associated symptoms were monitored during each visit. Nasopharyngeal and oropharyngeal swabs were taken at all three visits to identify the pathogen causing the ILI symptoms by qPCR. Cases of influenza-A virus were detected by real time PCR-based Multiplex Ligation-dependent Probe Amplification (MPLA) (RespiFinder^®^ Smart 22 kit, Pathofinder). All participants included in this study were confirmed CMV-seronegative as determined by ELISA (26) or our in-house Multiplex Immunoassay (27).

### PBMC Isolation and Flow Cytometry

Peripheral Blood Mononuclear cells (PBMCs) were isolated by density gradient (Ficoll-Hypaque, Amersham Biosciences) from heparinized blood or buffy coats and stored at -135°C in 10% dimethyl sulfoxide (DMSO, Sigma Aldrich) and fetal calf serum (FCS) until further use. For flow cytometric analyses, frozen PBMCs were thawed at 37°C and were transferred to RPMI-1640 medium (GIBCO, Thermo Fisher Scientific) supplemented with 10% FCS and Penicillin-Streptomycin-Glutamine (P/S/G) and washed twice. Thawed cells were then rested in medium for 30 minutes at room temperature after which the number of viable cells was determined on a Coulter Counter (Beckman). 4*10^5^ PBMCs of each individual were labeled for surface markers at 4°C for 30 minutes in FACS buffer (1x PBS +0.5% BSA +2mM EDTA) with saturating concentrations of the following fluorescent labeled antibodies: CD3-FITC (clone UCHT1), CD4-PerCP-Cy5.5 (clone RPA-T4), CD28-BV711 (clone CD28.2), CD27-BV510 (clone O323), and CD57 (clone HCD57) all from Biolegend, CD8-BUV395 (clone RPA-T8, BD Horizon), TIGIT-PE-eFluor610 (clone MBSA43, eBioscience), CD226-BV785 (clone DX11, BD Optibuild), and KLRG1-PerCP-eFluor710 (clone 13F12F2, Invitrogen). A Fixable Viability Stain 780 (BD Horizon) was added to the labeling to identify viable cells. Cells were then fixed and permeabilized with buffers for intracellular labeling (eBioscience) at 4°C for 30 minutes with fluorescent labeled antibodies targeting: Helios-PE-Cy7 (clone 22F6, Biolegend) and γH2AX (clone N1-431, BD Horizon). For *ex vivo* analyses on influenza virus antigen-specificity of CD8^+^ T cells in HLA-A2^+^ influenza A patients, 2-6*10^6^ PBMCs were first labeled with an HLA-A2 GILG-dextramer (A*0201/GILGFVFTL dextramer, FITC-conjugated, Immudex) for 20 minutes at room temperature before extra- and intracellular labeling as described above. We used fluorescence minus one (FMO) controls for all relevant markers and included these in our analyses to distinguish between positive and negative cells. Samples were measured on an LSRFortessa™ X-20 (BD Biosciences) and data were analyzed using FlowJo software (v10.6.1, TreeStar). The gating strategy used to define T-cell subsets is shown in **Supplementary Figure 3**.

### T-Cell Stimulation Assays

The number of viable cells in thawed PBMC samples was determined manually using trypan blue staining and Bürker-Türk. Cells were then washed with PBS and labeled with 0.5 µM CellTrace™ Violet (Invitrogen) in PBS per milliliter of cell suspension (10^6^ cells/mL) for 20 minutes at 37°C to track their proliferation. Ice-cold RPMI-1640 medium (+10% FCS, +P/S/G) was added and cells were rested at room temperature for 5 minutes. Cells were centrifuged at 400*g* for 5 minutes and washed with RPMI-1640 medium (+10% FCS, +P/S/G) three times. Part of the CellTrace-labeled PBMCs were cultured in the presence of 0.005 µg/mL plate-bound purified mouse anti-human CD3 (clone HIT3α, BD Biosciences) in RPMI-1640 medium in U-bottom plates (2*10^5^ cells/well), whereas another part was directly labeled with fluorescent labeled antibodies (day zero). For each donor, expression of activation markers was assessed at day zero, day one, and day three after culturing. T-cell proliferation was assessed by dilution of CellTrace after three days of culturing. At each time point, cells were labeled for surface markers with the following fluorescent labeled antibodies: anti-CD3-FITC (clone UCHT1, at day zero), anti-CD4-PerCP-Cy5.5 (clone RPA-T4), anti-CD69-BV785 (clone FN50), anti-CD25-PE (clone M-A251) all from Biolegend, anti-CD8-BUV395 (clone RPA-T8, BD Horizon), anti-TIGIT-PE-eFluor610 (clone MBSA43, eBioscience), and anti-CD226-BV785 (clone DX11, BD Optibuild). A Fixable Viability Stain 780 (BD Horizon) was added to the labeling to identify viable cells. Cells were labeled for intracellular markers with the following fluorescent labeled antibodies: anti-CD3-FITC (clone UCHT1, Biolegend, at days one and three) and anti-Helios-PE-Cy7 (clone 22F6, Biolegend).

### Intracellular Cytokine Assay

PBMCs were added to U-bottom plates (1*10^6^ cells/well) in RPMI-1640 medium and exposed to phorbol-12-myristate-13-acetate (PMA, 50 ng/mL, Sigma-Aldrich) with ionomycin (1 μg/mL, Sigma-Aldrich) for 6 hours at 37°C or to medium only as control. After two hours of incubation, Protein Transport Inhibitor Brefeldin A (BD GolgiPlug; 1:1000) was added and present during the final four-hour incubation period to promote intracellular accumulation of cytokines. After incubation, cells were labeled for surface makers for 30 minutes at 4°C with saturating concentrations of fluorescent-labeled antibodies: anti-CD3-FITC (clone UCHT1, Biolegend), anti-CD8-BUV395 (clone RPA-T8, BD Horizon), anti-TIGIT-PE-eFluor610 (clone MBSA43, eBioscience) in FACS buffer. Fixable Viability Stain 780 (BD Horizon) was added during the surface marker staining to identify viable cells. Surface staining was followed by fixation and permeabilization and intracellular labeling using anti-Helios-PE-Cy7 (clone 22F6), anti-IL-2-BV785, anti-IL-10-BV421 and anti-TNF-α-PerCP-Cy5.5 (clone Mab11) from Biolegend, and anti-IFN-γ-BUV737 (4S.B3, BD Pharmingen) using buffers of the eBioscience Foxp3-transccription factor staining buffer set. Frequencies of cells expressing PMA/ionomycin-induced cytokines were calculated for each of the cytokines per specimen by substracting % of cytokine producing cells from % of cytokine producing cells cultured with PMA and ionomycin. Samples were measured on an LSRFortessa™ X-20 (BD Biosciences) and data were analyzed using FlowJo software (v10.6.1, TreeStar).

### Dimensionality Reduced Analyses (viSNE)

Dimensionality reduced analysis (viSNE) of flow cytometry data was performed in Cytobank (http://www.cytobank.org) (28). Fingerprint color dot plots generated by viSNE indicate expression of each of the indicated markers within the CD8^+^ T-cell population as measured by flow cytometry. These plots were generated based on measurements in one donor to illustrate the overlap of markers within CD8^+^ T cells. A number of 6,325 CD8^+^ T cells was included in this analysis.

### Statistics

Statistical analysis was performed using GraphPad Prism version 8.4.1. The appropriate parametric or non-parametric tests were used based on the tested normality of distribution of the data. Paired analyses were performed with non-parametric Friedman Test with Dunn’s post-test. Correlations between variables were analyzed using Spearman’s rank correlation coefficient (*r*). Linear regression analysis was performed to generate lines of best fit. Statistical significance was considered when *p* < 0.05, with *p <0.05, **p <0.01, ***p <0.001, and ****p <0.0001. All data presented in bar graphs are depicted as mean ± s.d.

## Results

### Co-Expression of TIGIT and Helios Defines Functionally Senescent CD8^+^ T Cells

**Figure 1:**
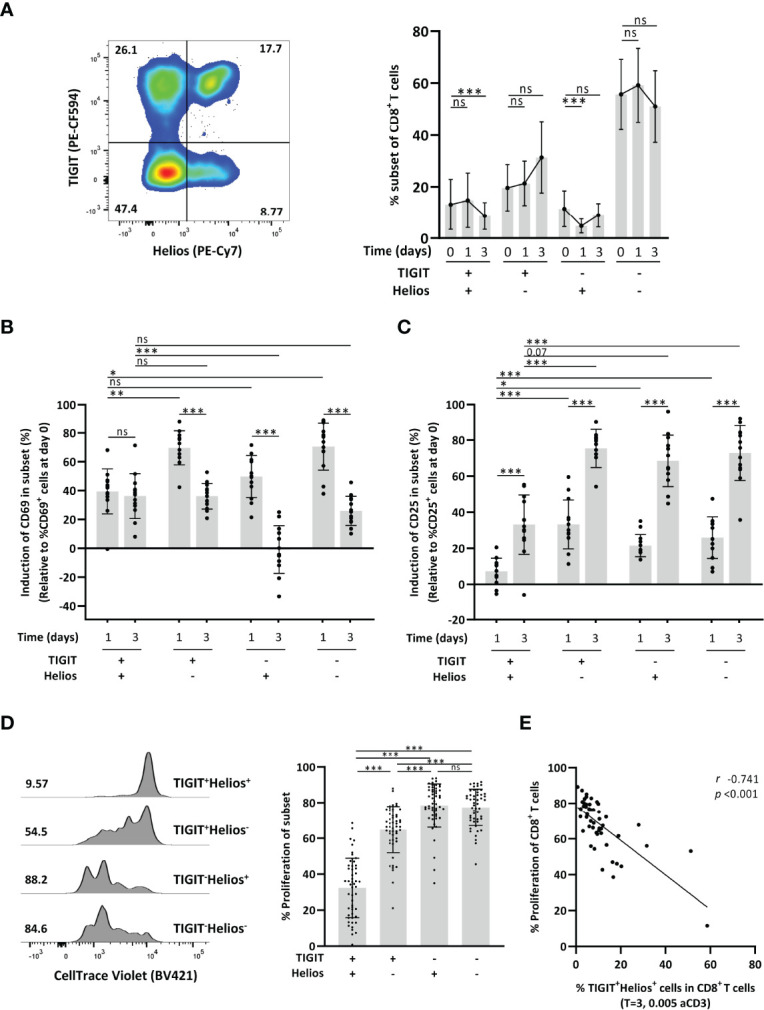
Co-expression of TIGIT and Helios defines functionally senescent CD8^+^ T cells. **(A)** Representative flow-cytometry plot with expression of TIGIT and Helios by CD8^+^ T cells and the frequency of TIGIT/Helios cell subsets among CD8^+^ T cells over time after stimulation of unsorted whole PBMC samples with anti-CD3 (day zero, one, and three) are shown. Induction of T-cell activation was assessed by measuring the frequency of **(B)** CD69^+^ cells and **(C)** CD25^+^ cells over time within the indicated TIGIT/Helios cell subsets relative to the frequency of CD69^+^ and CD25^+^ cells determined at day zero (% CD69^+^ or CD25^+^ cells at indicated day minus their % at day 0) (n = 13). T-cell proliferation was assessed by dilution of CellTrace after three days of stimulation with anti-CD3. **(D)** Representative histogram shows the proliferation of the indicated TIGIT/Helios cell subsets by dilution of CellTrace (number indicates the frequency of proliferated cells of the subset). Bar graph shows the frequency of proliferated cells within each TIGIT/Helios cell subset (n = 50). **(E)** Relationship between the frequency of proliferated CD8^+^ T cells and the frequency of TIGIT^+^ Helios^+^ cells within the CD8^+^ T-cell population after three days of stimulation. All frequencies of proliferating cells were relative to their unstimulated control sample. Correlations (*r* and *p* values) were assessed by Spearman test. Statistical significance of data presented in the bar graphs (means ± s.d.) was determined using Friedman test (with Dunn’s post-test). Wilcoxon test was used in panels **(B, C)** for the comparison between the time points within each subset. (*p < 0.05, **p < 0.01, ***p < 0.001, ns, not significant).

We first questioned if co-expression of TIGIT and Helios links to dysfunctional immunosenescent CD8^+^ T cells. We therefore explored if co-expression of TIGIT and Helios (**Figure 1A**) would relate to reduced capacity of CD8^+^ T cells to get activated and proliferate in response to *in vitro* stimulation of PBMCs with anti-CD3. The proportion of TIGIT^+^Helios^+^ T cells within the CD8^+^ T-cell population remained stable after one day of culturing with anti-CD3, although this proportion declined after three days (**Figure 1A**). TIGIT^+^Helios^+^ cells were activated within the first day of culture (shown by induction of classic activation markers CD69 and CD25), but to a lesser extent than the TIGIT^+^Helios^-^ and TIGIT^-^Helios^-^ cell subsets (**Figures 1B, C** and **Supplementary Figure 1**). Two days later, expression of the early activation marker CD69 had significantly dropped in the cells that were single positive or double negative for TIGIT and Helios. Such rapid return towards lower CD69 expression during a response *in vitro* is typically observed after activation of T cells (7). However, TIGIT^+^Helios^+^ cells did not show a decline of CD69 expression, but the induced CD69 expression appeared to persist on these cells instead (**Figure 1B**). From day 1 to day 3, expression of the activation marker CD25 increased further in all four subsets, but this upregulation was lower in TIGIT^+^Helios^+^ cells (**Figure 1C**). Together, these data indicate reduced activation potential of CD8^+^ T cells co-expressing TIGIT and Helios.

Although all TIGIT^+^ subsets showed reduced proliferation, TIGIT^+^ cells co-expressing Helios proliferated less compared to all other subsets (**Figure 1D**). In addition, the proportion of the TIGIT^+^Helios^+^ subset within the CD8^+^ T-cell population negatively correlated with proliferation of the total CD8^+^ T cell population (**Figure 1E**), indicating a significant contribution of this TIGIT^+^Helios^+^ subset to decline in proliferative capacity of the overall CD8^+^ T-cell population.

**Figure 2:**
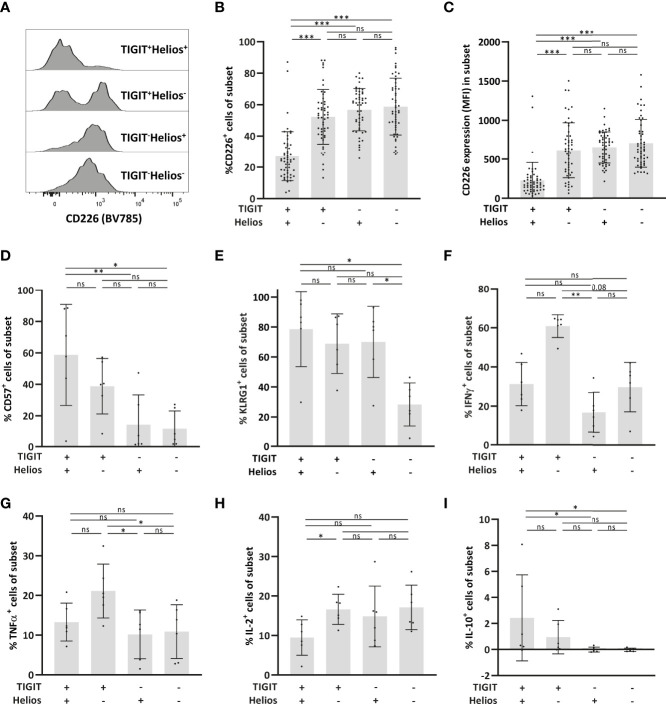
Low expression of the co-stimulatory receptor CD226 by immunosenescent CD8^+^ T cells. **(A)** Representative histogram of CD226 expression within each of the TIGIT/Helios cell subsets. Bar graphs show **(B)** the frequency of CD226^+^ cells and **(C)** expression (median fluorescent intensity, MFI) of CD226 per cell within each of the four TIGIT/Helios cell subsets (n = 50). Bar graphs show the frequency of **(D)** CD57^+^ and **(E)** KLRG1^+^ cells within each of the four TIGIT/Helios cell subsets (n = 6). Bar graphs show the frequency of **(F)** IFN-γ^+^, **(G)** TNF-α^+^, **(H)** IL-2^+^, and **(I)** IL-10^+^ cells within each of the four TIGIT/Helios cell subsets in response to PMA/ionomycin. Statistical significance of data presented in the bar graphs (means ± s.d.) was determined using Friedman test (with Dunn’s post-test). (*p < 0.05, **p < 0.01, ***p < 0.001, ns, not significant).

The co-stimulatory receptor CD226 is the counterpart of TIGIT (29) and lack of CD226 expression has recently been shown to mark senescent T cells in mice (30). TIGIT^+^Helios^+^ cells showed reduced expression of this marker (**Figures 2A-C**). In a small subset of samples, we performed additional flowcytometry to explore other immunosenescence-related features in these cells. Senescence-associated markers CD57 and killer cell lectin-like receptor G1 (KLRG1) (31, 32) were highly expressed by TIGIT^+^Helios^+^ cells (**Figures 2D, E**). TIGIT^+^Helios^+^ cells showed higher frequencies of CD57^+^ cells compared to TIGIT^-^ cells, irrespective of Helios expression (**Figure 2D**). For KLRG1, higher frequencies were observed within the TIGIT^+^Helios^+^ subset compared to the double negative subset, but not the other subsets (**Figure 2E**). Lastly we analyzed production of cytokines in response to PMA+ionomycin. Whereas TIGIT^+^Helios^+^ cells did not show different frequencies of intracellular IFN-γ^+^ and TNF-α^+^ cells (**Figures 2F, G**), the frequency of IL-2^+^ cells was lower compared to TIGIT^+^Helios^-^ cells (**Figure 2H**). Additionally, the frequency of IL-10^+^ cells amongst TIGIT^+^Helios^+^ cells was higher compared to TIGIT^-^Helios^+^ and TIGIT^-^Helios^-^ cells (**Figure 2I**), but it comprised only a minor fraction of this T cell population.

Overall, these findings show that Helios more accurately defines immunosenescence among the previously reported TIGIT^+^ CD8+ T-cell population that increases with age (15). Therefore, co-expression of TIGIT and Helios can be used to define functionally senescent CD8^+^ T cells.

### TIGIT^+^Helios^+^ CD8^+^ T Cells Are Highly Activated During Influenza-A Infection and Correlate With Prolonged Coughing

Viral respiratory infections such as influenza-A infection especially cause higher morbidity and mortality at older age (2). To explore a potential clinical role of TIGIT^+^Helios^+^ CD8^+^ T cells, we analyzed their presence and activation status in the peripheral blood of older adults suffering from an acute influenza-A infection (63-83 years of age, n=15; H3N2 n=12, H1N1 n=3) obtained at the acute phase of influenza and during follow-up at two and eight weeks.

**Figure 3:**
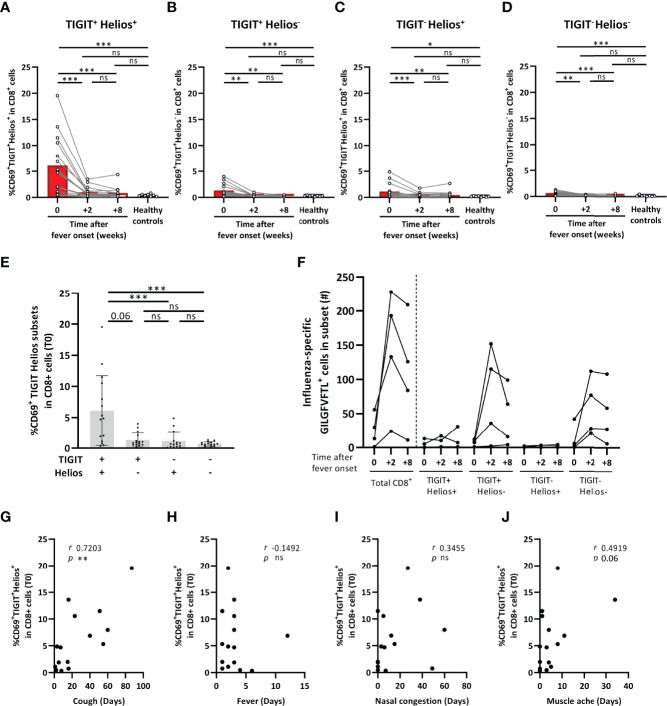
TIGIT^+^Helios^+^ CD8^+^ T cells are highly activated during influenza-A infection and correlate with prolonged coughing. **(A–D)** Bar graphs show the frequency of CD69^+^ TIGIT/Helios cell subsets within the total CD8^+^ T-cell population as detected in blood of older adults at the acute phase of influenza with confirmed influenza-A infection (0), and two (+2) and eight (+8) weeks after onset of fever (63-83 years of age, n = 15; H3N2 n = 12, H1N1 n = 3), as well as in older adult asymptomatic controls (61-82 years of age, n=8). **(E)** Bar graph shows the frequency of CD69^+^ TIGIT/Helios cell subsets within the total CD8^+^ T-cell population in influenza-A infected individuals at the acute phase of influenza. **(F)** Graph shows the number of cells specific for the GILGFVFTL influenza A matrix protein epitope at the indicated time after onset of fever within the total CD8^+^ T-cell population and the TIGIT/Helios cell subsets (n = 4). Relationship between the frequency of CD69^+^TIGIT^+^Helios^+^ cells within the total CD8^+^ T-cell population and duration of **(G)** cough, **(H)** fever, **(I)** nasal congestion, and **(J)** muscle ache. Correlations (*r* and *p* values) were assessed by Spearman test. Statistical significance of data presented in the bar graphs (means ± s.d.) was determined using row-matched one-way ANOVA (with Geisser-Greenhouse correction and Dunnett’s post-test) for the difference between the time points and Mann-Whitney *U* test was used to determine the difference between infected and asymptomatic individuals. (*p < 0.05, **p < 0.01, ***p < 0.001, ns, not significant).

The proportion of TIGIT^+^Helios^+^ T cells within the total CD8^+^ T-cell population slightly declined from acute disease to two and eight weeks after symptom onset, although proportions at each time point did not differ from those found in older adult asymptomatic controls (**Supplementary Figure 2**). However, all TIGIT/Helios cell subsets showed significantly elevated frequencies of activated cells by CD69 expression at the acute phase of disease (**Figures 3A–D**) that declined at later time points, indicating that all subsets contain cells that are activated by influenza virus infection. Remarkably, activation of CD8^+^ T cells was most profound in the TIGIT^+^Helios^+^ subset (**Figure 3E**). Additionally, we assessed the number of influenza-specific T cells in HLA-A2^+^ individuals (n=4) within the total CD8^+^ T-cell population and within the TIGIT/Helios subsets by labelling these cells with dextramers containing the immunodominant influenza A epitope GILGFVFTL (33). The number of influenza-A specific TIGIT^+^Helios^+^ was relatively low in all 4 donors, suggesting that the majority of activated TIGIT^+^Helios^+^ cells are not influenza specific. (**Figure 3F**).

Lastly, we investigated whether the high proportion of activated TIGIT^+^Helios^+^ cells at the onset of influenza correlated with self-reported influenza-A-associated symptom duration. The proportion of CD69^+^TIGIT^+^Helios^+^ cells within the total CD8^+^ T-cell population showed a strong positive relationship with the duration of coughing (*r* 0.7203; *p* 0.0033) (**Figure 3G**), whereas none of the other TIGIT/Helios cell subsets did (**Supplementary Figure 2**). The proportion of CD69^+^TIGIT^+^Helios^+^ cells did not show relationships with the duration of other symptoms such as fever and nasal congestion, aside from a trend for a positive relationship with muscle ache (*r* 0.4919; *p* 0.06) (**Figures 3H–J**). Together, these data suggest a potential disease-prolonging role for TIGIT^+^Helios^+^ cells during influenza-A infection.

### Late-Differentiated CD8^+^ T Cells Accumulate With Age at the Cost of Early-Differentiated T Cells

**Figure 4:**
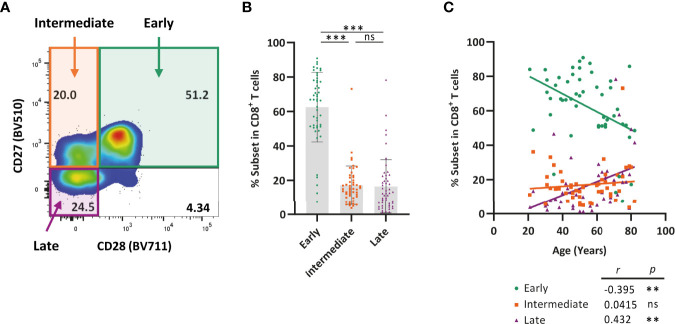
Late-differentiated T cells accumulate with age at the cost of early-differentiated T cells. Expression of CD27 and CD28 by CD8^+^ T cells was analyzed in healthy individuals ranging from 21-82 years of age (n = 50) using flow cytometry. **(A)** Representative flow-cytometry plot illustrating expression of CD27 and CD28 by CD8^+^ T cells. **(B)** Bar graphs show the frequency of early (CD27^+^CD28^+^), intermediate (CD27^+^CD28^-^), and late (CD27^-^CD28^-^) differentiated cells within the total CD8^+^ T-cell population of each individual. **(C)** Frequency of each of the three differentiation subsets among CD8^+^ T cells and their relationship with age. Correlations (*r* and *p* values) were assessed by Spearman test. Statistical significance of data presented in the bar graph (means ± s.d.) was determined using Friedman test (with Dunn’s post-test). (**p < 0.01, ***p < 0.001, ns, not significant).

We next explored how expression of TIGIT and Helios relates to age and to the differentiation status of CD8^+^ T cells. To do so, we first analyzed differentiation status of the cells in the subjects in our cohort. CD8^+^ T cells are known to downregulate CD27 and CD28 expression during aging by transition from CD27^+^CD28^+^ early-differentiated cells to CD27^+^CD28^-^ intermediate-differentiated cells and, finally, into CD27^-^CD28^-^ late-differentiated T cells leading to accumulation of the latter subset during aging (10, 13, 14, 34). We found that early-differentiated cells were most abundant amongst CD8^+^ T cells in comparison to the presence of intermediate- and late-differentiated cells (**Figures 4A, B**). With age, the proportion of late-differentiated cells increased, whereas the proportion of early-differentiated cells declined (**Figure 4C**). The proportion of intermediate-differentiated cells did not change with age. These findings show that late-differentiated cells accumulate with age at the expense of early-differentiated cells.

### TIGIT^+^Helios^+^ Cells Are Enriched in the Intermediate‐ and Late‐Differentiated CD8^+^ T‐Cell Populations

**Figure 5:**
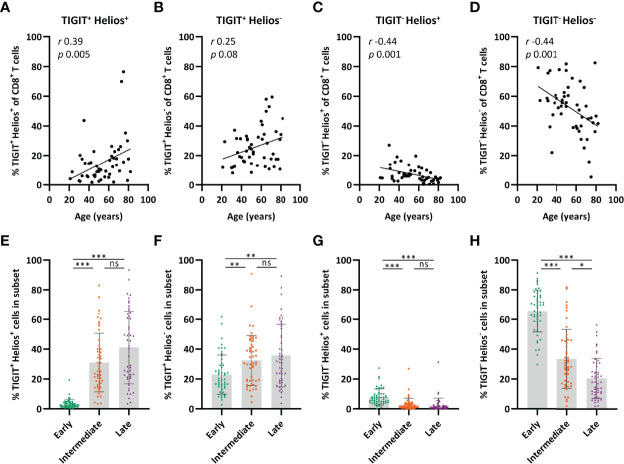
TIGIT^+^ Helios^+^ cells are enriched in the intermediate- and late-differentiated CD8^+^ T-cell populations. Relationship between the frequency of **(A)** TIGIT^+^ Helios^+^, **(B)** TIGIT^+^ Helios^-^, **(C)** TIGIT^-^ Helios^+^, and **(D)** TIGIT^-^ Helios^-^ cells within the CD8^+^ T-cell population and age (n = 50). Bar graphs show the frequency of **(E)** TIGIT^+^ Helios^+^, **(F)** TIGIT^+^ Helios^-^, **(G)** TIGIT^-^ Helios^+^, and **(H)** TIGIT^-^ Helios^-^ cells within the early-, intermediate-, and late-differentiated cell subsets. Correlations (*r* and *p* values) were assessed by Spearman test. Statistical significance of data presented in the bar graphs (means ± s.d.) was determined using Friedman test (with Dunn’s post-test). (*p < 0.05, **p < 0.01, ***p < 0.001, ns, not significant).

Clustering of CD8^+^ T cells based on their expression of CD27, CD28, TIGIT, and Helios by viSNE analysis indicated that only part of the CD8^+^ T-cell pool co-expresses TIGIT and Helios and that expression of these molecules may relate to distinctive expression of the differentiation markers CD27 and CD28 (**Supplementary Figure 3**). The proportion of TIGIT^+^Helios^+^ cells amongst total CD8^+^ T cells increased at older age (**Figure 5A**) and the proportion of TIGIT^+^Helios^-^ cells showed a trend towards higher frequencies with age (**Figure 5B**). The increase of both these subsets appeared to be at the expense of TIGIT^-^Helios^+^ and TIGIT^-^Helios^-^ proportions within the total CD8^+^ T-cell population (**Figures 5C, D**). Notably, TIGIT^+^Helios^+^ cells appeared to be present mostly in the intermediate- and late-differentiated cell subsets (**Figure 5E**), which could also be observed for TIGIT^+^Helios^-^ cells, although to a lesser extent (**Figure 5F**). Both TIGIT^-^Helios^+^ and TIGIT^-^Helios^-^ cells were mostly present within the early-differentiated subset (**Figures 5G, H**). Together, these findings indicate that co-expression of TIGIT and Helios defines an aging-related population of immunosenescent cells that accumulate in the intermediate- and late-differentiated CD8^+^ T-cell subsets. Since our study focuses on aging-related senescence, we also analyzed if the TIGIT^+^Helios^+^ population would increase with age within the CD4^+^ T cell compartment. We observed no correlation (*r*=0.15; *p*=0.30) of this phenotype among CD4^+^ T cells with older age (data not shown).

### Progression of CD8^+^ T-Cell Senescence (TIGIT^+^Helios^+^) During Aging Occurs Already in the Intermediate Phase of Differentiation

**Figure 6:**
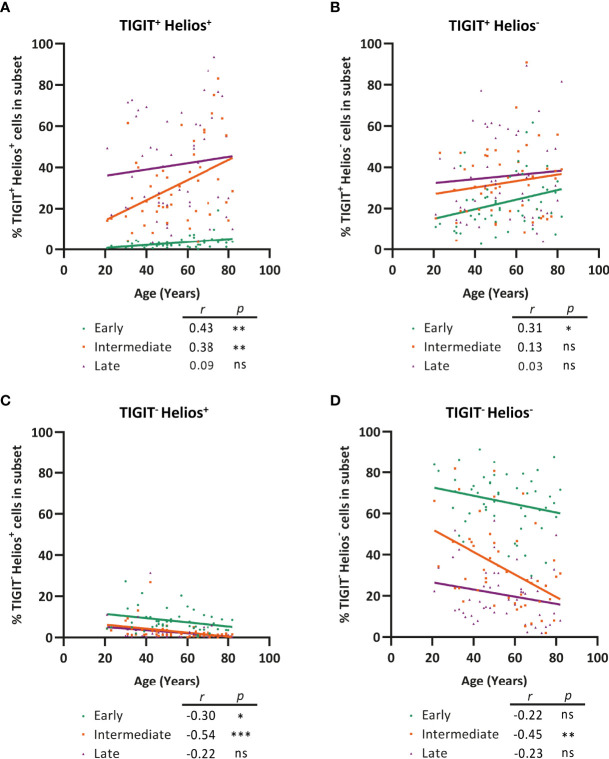
Progression of CD8^+^ T-cell senescence (TIGIT^+^ Helios^+^) during aging occurs already in the intermediate phase of differentiation. Frequency of **(A)** TIGIT^+^ Helios^+^, **(B)** TIGIT^+^ Helios^-^, **(C)** TIGIT^-^ Helios^+^, and **(D)** TIGIT^-^ Helios^-^ cells within the early-, intermediate-, and late-differentiated cell subsets and their relationship with age (n = 50). Correlations (*r* and *p* values) were assessed by Spearman test with *p < *0.05 considered as statistically significant. (*p < 0.05, **p < 0.01, ***p < 0.001, ns, not significant).

We next assessed whether the frequency of cells expressing TIGIT and Helios within early-, intermediate-, and late-differentiated CD8^+^ T-cell subsets changes with age. Interestingly, the frequency of TIGIT^+^Helios^+^ cells increased significantly within the intermediate-differentiated CD8^+^ T cells subset with age, but not so within the late-differentiated subset (**Figure 6A**). Moreover, despite age-related decline of the fraction of early-differentiated cells (**Figure 4C**), the fraction of TIGIT^+^Helios^+^ cells among the early-differentiated population increases with age (**Figure 6A**). These findings show that a significant fraction of senescent cells as defined by TIGIT^+^Helios^+^ already accumulates in the intermediate-differentiated CD8^+^ T-cell population at older age. In addition, the frequency of TIGIT^+^Helios^-^ cells only increases within the early-differentiated subset (**Figure 6B**). Moreover, the rise of TIGIT^+^Helios^+^ cell frequencies appear to be at the expense of declining frequencies of TIGIT^-^Helios^+^ and TIGIT^-^Helios^-^ cells within the intermediate-differentiated subset (**Figures 6C, D**). Thus, these findings suggest that CD8^+^ T cells develop into immunosenescent TIGIT^+^Helios^+^ cells during the course of aging already before reaching the late stage of differentiation.

### TIGIT^+^Helios^+^ Intermediate- and TIGIT^+^Helios^+^ Late-Differentiated Cells Accumulate With Age Within the CD8^+^ T-Cell Population at the Cost of TIGIT^-^Helios^+^ and TIGIT^-^Helios^-^ Cells

**Figure 7:**
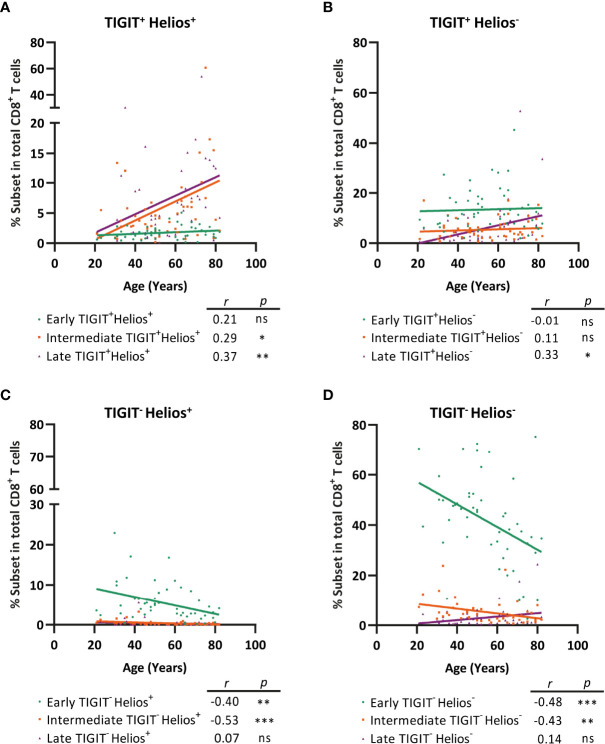
TIGIT^+^ Helios^+^ intermediate- and TIGIT^+^Helios^+^ late-differentiated cells accumulate with age within the total CD8^+^ T-cell population at the cost of TIGIT^-^ Helios^+^ and TIGIT^-^ Helios^-^ cells. Frequency of early-, intermediate-, or late-differentiated **(A)** TIGIT^+^ Helios^+^ cells, **(B)** TIGIT^+^ Helios^-^ cells, **(C)** TIGIT^-^ Helios^+^ cells, and **(D)** TIGIT^-^ Helios^-^ cells within the total CD8^+^ T-cell population and their relationship with age (n = 50). Correlations (*r* and *p* values) were assessed by Spearman test with *p < *0.05 considered as statistically significant. (*p < 0.05, **p < 0.01, ***p < 0.001, ns, not significant).

Lastly, we addressed whether early-, intermediate-, and late-differentiated cells expressing TIGIT and/or Helios increase by aging within the total CD8^+^ T-cell population. Interestingly, not only the frequency of TIGIT^+^Helios^+^ late-differentiated cells increased amongst the total CD8^+^ T-cell population with age, but also the frequency of TIGIT^+^Helios^+^ intermediate-differentiated cells (**Figure 7A**). In analyses of TIGIT^+^ cells not expressing Helios, only late-differentiated TIGIT^+^Helios^-^ cells accumulate within the CD8^+^ T-cell population with age (**Figure 7B**). This indicates that Helios in addition to TIGIT serves as marker for the identification of senescent cells among the intermediate-differentiated population. Furthermore, the increase of senescent CD8^+^ T cells with age appears to be at the cost of TIGIT^-^Helios^+^ and TIGIT^-^Helios^-^ T cells both as early- and intermediate-differentiated cells (**Figures 7C, D**). Together, these findings indicate that TIGIT^+^Helios^+^ T cells accumulate in older adults not only as late-differentiated cells but also as intermediate-differentiated cells within the total CD8^+^ T-cell population.

## Discussion

The accumulation of late-differentiated CD8^+^ T cells at older age is widely used as a marker of immunological aging due to features of immunosenescence (11, 14). However, focusing on the late differentiation stage may neglect occurrence of immunosenescence of CD8^+^ T-cells in earlier stages of differentiation. We here identified co-expression of TIGIT and Helios as an more accurate hallmark of immunosenescent CD8^+^ T cells. We show that these immunosenescent cells accumulate with age not only by increase of the late-differentiated population, but also by aging-related development of functional features of immunosenescence already within earlier stages of differentiation. Therefore, accumulation of functionally immunosenescent cells at old age is partly independent of the final progression into late stage of differentiation. Moreover, we show that TIGIT^+^Helios^+^ T cells are highly activated during influenza and correlate with prolonged coughing. These findings indicate that analysis of aging-related development of immunosenescence should include functional hallmarks of immunosenescence such as TIGIT and Helios.

TIGIT has recently been identified as a marker for immunosenescent CD8^+^ T cells that accumulate at older age (15–17). By including Helios in our analyses, we found that the TIGIT^+^ T-cell population is heterogeneous and that only a part of the TIGIT^+^ T cells co-expressed Helios. Particularly the TIGIT^+^Helios^+^ T-cell subset showed features of immunosenescence identified by low capacity to proliferate and become activated as well as low expression of the co-stimulatory receptor CD226, trends towards lower IL-2 production, and high frequencies of senescence-associated markers CD57 and KLRG1. These findings indicate that Helios more accurately defines immunosenescent cells among the TIGIT^+^ CD8^+^ T-cell population reported on previously (15). In addition, late-differentiated senescent T cells have been reported to produce high levels of IFN-γ and TNF-α (10). However, we found that production of these cytokines by the TIGIT^+^Helios^+^ subset of CD8^+^ T cells was not higher than in the other subsets. TIGIT^+^Helios^+^ cells do not fully match the classical cytokine-profile of late-differentiated T cells, whereas only TIGIT^+^Helios^-^ cells slightly resembled the cytokine profile with higher IFN-γ and TNF-α levels. These findings on the addition of Helios as marker indicate that the TIGIT^+^ CD8^+^ T-cell population that has recently been defined as immunosenescent population is heterogeneous (15). Including analysis of Helios expression may therefore advance future studies on TIGIT^+^ CD8^+^ T cells in immunosenescence. Our finding that predominantly the Helios^+^ subset of TIGIT^+^ cells comprises immunosenescent cells also implies that interventions to overcome immunosenescence by targeting the co-inhibitory receptor TIGIT (16, 35) may be refined by particularly targeting this most immunosenescent subset rather than all TIGIT^+^ CD8^+^ T cells. Moreover, our findings warrant exploration of TIGIT^+^Helios^+^ CD8^+^ T cells with respect to other known markers of aging and immunosenescence in CD8^+^ T cells, such as NK cell-related receptors, and low telomerase expression (10, 36).

TIGIT^+^Helios^+^ T cells are highly activated during the acute phase of influenza infection and this activation showed a strong positive relationship with the duration of flu-associated coughing. Moreover, the majority of these cells did not appear to be influenza-specific, indicating that these cells may be bystander cells during viral respiratory infection. As such, a likely explanation for T cells responding not influenza-antigen specifically is that these cells are activated as bystander cells responding to cytokines produced by influenza antigen-specific T cells or by innate stimuli derived from the virus infection. *Via* this way, these T cells may control or contribute to the overall T-cell response at the time of a virus infection. Prolonged presence of coughing may indicate reduced viral clearance from the lungs (37), hinting towards a disease-prolonging effect of TIGIT^+^Helios^+^ T cells. As CD8^+^ T cells are vital for viral clearance from the lungs (38–41) and as we show that TIGIT^+^Helios^+^ are phenotypically and functionally senescent, these cells may negatively affect viral clearance and recovery from an influenza-A infection. On the other hand, our clinical data do not exclude that activated TIGIT^+^Helios^+^ T cells may contribute to preventing severe disease. Such function is highlighted by the recently discovered role for expression of TIGIT in ameliorating acute disease induced by lymphocytic choriomeningitis virus (LCMV) and influenza virus in mice by preventing overactivation of CD8^+^ T cells and reducing tissue damage (42). It remains to be established whether or not these TIGIT^+^ T cells express Helios. In addition to such regulatory role of TIGIT in LCMV and influenza, some recent studies have shown that TIGIT and Helios are expressed by subsets of CD8^+^ regulatory T cells and contribute to their suppressive function (43, 44). Therefore, it is likely that the activated TIGIT^+^Helios^+^ T cells we identified may have an immunosuppressive function during infection. Thus, our findings indicate clinical relevance for senescent TIGIT^+^Helios^+^ T cells to serve as a potential correlate of disease during an acute viral respiratory infection and warrant further investigation into this cell population.

We observed the association between activated TIGIT^+^Helios^+^ and prolonged cough in a modest cohort size and the statistics may potentially be modulated by the late follow up to 90 days for one donor. The non-parametric approach we used to analyze the samples from the influenza cohort is robust to such outliers and would have circumvented disproportional impact of samples showing extremer values on overall significance. Moreover, analyzing this set of data after excluding this outlier, we still found a significant correlation between longer duration of cough and higher frequency of activated TIGIT^+^Helios^+^ cells (*r*=0.6557; *p*=0.0129).

We found that the proportion of late-differentiated T cells rises with age and is highly enriched with immunosenescent cells as defined by TIGIT^+^Helios^+^. This indicates that the CD8^+^ T-cell pool becomes enriched with these immunosenescent cells over the years. Previous work has reported that CD8^+^ T cells follow a linear differentiation pathway from early- to late-differentiated state through the intermediate-differentiated phase (13). Interestingly, although the proportion of cells in the intermediate-differentiation state did not alter with age, we found that this intermediate CD8^+^ T-cell population becomes enriched with immunosenescent TIGIT^+^Helios^+^ cells at older age. Moreover, the proportion of TIGIT^+^Helios^+^ intermediate-differentiated cells among the CD8^+^ T-cell population increased with age. These findings indicate that CD8^+^ T cells may become immunosenescent before entering the late-differentiation state and that aging-related increase of immunosenescence cannot merely be classified by accumulation of highly differentiated CD27^-^CD28^-^ cells. Interestingly, it has been shown that elevated numbers of intermediate-differentiated CD8^+^ T cells is a predictor of frailty (45), emphasizing the significance of this subset during aging. The applicability of differentiation state based on CD27 and CD28 expression as proxy for immunosenescence may thus depend on age. Our findings suggest that especially at older age, part of the intermediate-differentiated population may be regarded immunosenescent in addition to late-differentiated T cells. Hence, this indicates that differentiation and development of immunosenescence with age do not fully progress in parallel and warrants further research on factors that drive these two processes. In addition to such complexity of development of senescence with age, we observed that TIGIT^+^Helios^+^ does not fully overlap the CD27/CD28-expression profiles (**Supplementary Figure 3A**). Therefore, future studies allowing more in depth analyses of direct comparisons on the similarity and difference between the TIGIT^+^Helios^+^ and CD27/CD28-defined CD8 T cell subsets are needed to further improve understanding of definition of senescence using these markers.

Accumulation of CD27^-^CD28^-^ cells has often been ascribed to latent infection by cytomegalovirus (CMV) as a result of repetitive T-cell stimulation (13, 46, 47). Our results show that CD8^+^ T cells significantly lose expression of CD27 and CD28 and gain features of immunosenescence over the years in CMV seronegative individuals, indicating that expansion of highly differentiated T cells is not solely due to CMV. However, since latent CMV infection can promote differentiation of T cells it would be interesting to explore to what extent this contributes to the induction of immunosenescent TIGIT^+^Helios^+^ at old age.

A recent study suggested that Helios may play a role in immunosenescence of T cells (48) and our data now show that Helios expression clearly marks immunosenescence in CD8^+^ T cells along with co-expression of TIGIT. How Helios may contribute to altered function of CD8^+^ T cells at old age and how it becomes induced in the process of aging is unclear. Helios is known as a transcription factor that is expressed by regulatory T cells and needed for their suppressive function (18, 19). Since immunosenescent T cells have gained several features known for regulatory T cells such as Helios and TIGIT, it is tempting to hypothesize that altered functions of a T cell gained at old age may be represented by gain of immunosuppressive functions. Targeting Helios may be a way to investigate the importance of Helios expression in functionality of these TIGIT^+^Helios^+^ CD8^+^ T cells. As Helios is an intracellular transcription factor which impedes sorting of TIGIT^+^Helios^+^ T cells, targeting Helios in future studies with small molecule inhibitors or protein mimetics would be an alternative way to study the role of Helios expression. Additionally, it would be helpful to find markers on the cell surface that may either specifically result from Helios activity or be a specific inducer of Helios.

Together, our study indicates that co-expression of TIGIT and Helios provides a novel useful marker to better define immunosenescent CD8^+^ T cells. Moreover, we show that these cells already accumulate in the intermediate-differentiation stage in older individuals. These findings challenge the current dogma of late-differentiation stage as a sole proxy for T-cell immunosenescence. These insights may refine future interpretation of studies on impaired CD8^+^ T-cell mediated immunity to viral infections at old age.

## Data Availability Statement

The original contributions presented in the study are included in the article/**Supplementary Material**. Further inquiries can be directed to the corresponding author.

## Ethics Statement

The studies involving human participants were reviewed and approved by the ethical committee METC Noord-Holland: NVI-255 study (Netherlands Trial Register NTR2070) ILI-3 study (Netherlands Trial Register NTR4818). The patients/participants provided their written informed consent to participate in this study.

## Author Contributions

DP, NS, and TG designed the study. DP, NS, VK, RP, and TG performed the experiments. JB designed and facilitated the sample collection of clinical cohort studies. DP, NS, and TG analyzed the data. DP, NS, JW, JB, DB, and TG interpreted the data. DP and TG wrote the manuscript. DP, JW, JB, DB, and TG reviewed and edited the manuscript. All authors contributed to the article and approved the submitted version.

## Funding

This study was supported by the Dutch Ministry of Health, Welfare and Sport.

## Conflict of Interest

The authors declare that the research was conducted in the absence of any commercial or financial relationships that could be construed as a potential conflict of interest.

## Publisher’s Note

All claims expressed in this article are solely those of the authors and do not necessarily represent those of their affiliated organizations, or those of the publisher, the editors and the reviewers. Any product that may be evaluated in this article, or claim that may be made by its manufacturer, is not guaranteed or endorsed by the publisher.
