# Supplementary material for: Co-Expression of TIGIT and Helios Marks Immunosenescent CD8+ T Cells During Aging
Source: Front Immunol. 2022 May 16;13:833531. doi: 10.3389/fimmu.2022.833531 (PMC9148977; doi:10.3389/fimmu.2022.833531)
Supplement: Supplementary file 1 [file DataSheet_1.docx]

***Supplementary Figures***

**
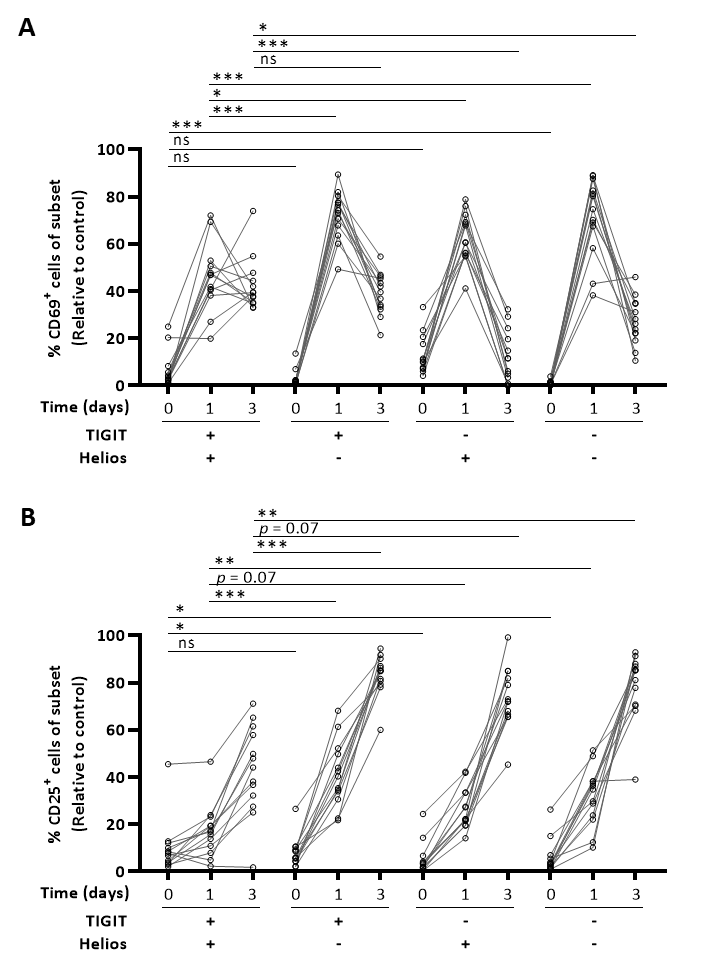
**

**Supplementary Figure 1. Kinetics of activation (CD69 and CD25) in TIGIT/Helios cell subsets.**

Total PBMCs were cultured in the presence of anti-CD3 for one or three days. Frequencies of (**A**) CD69^+^ cells and (**B**) CD25^+^ cells within the indicated TIGIT/Helios cell subsets (n=13) were determined before (day zero) and after stimulation (day one and three). For day one and three after stimulation, frequencies are depicted as relative to their unstimulated control sample. Statistical significance of data presented in the line graphs was determined using Friedman test (with Dunn’s post-test). (*p <0.05, **p <0.01, ***p <0.001, ns=not significant).


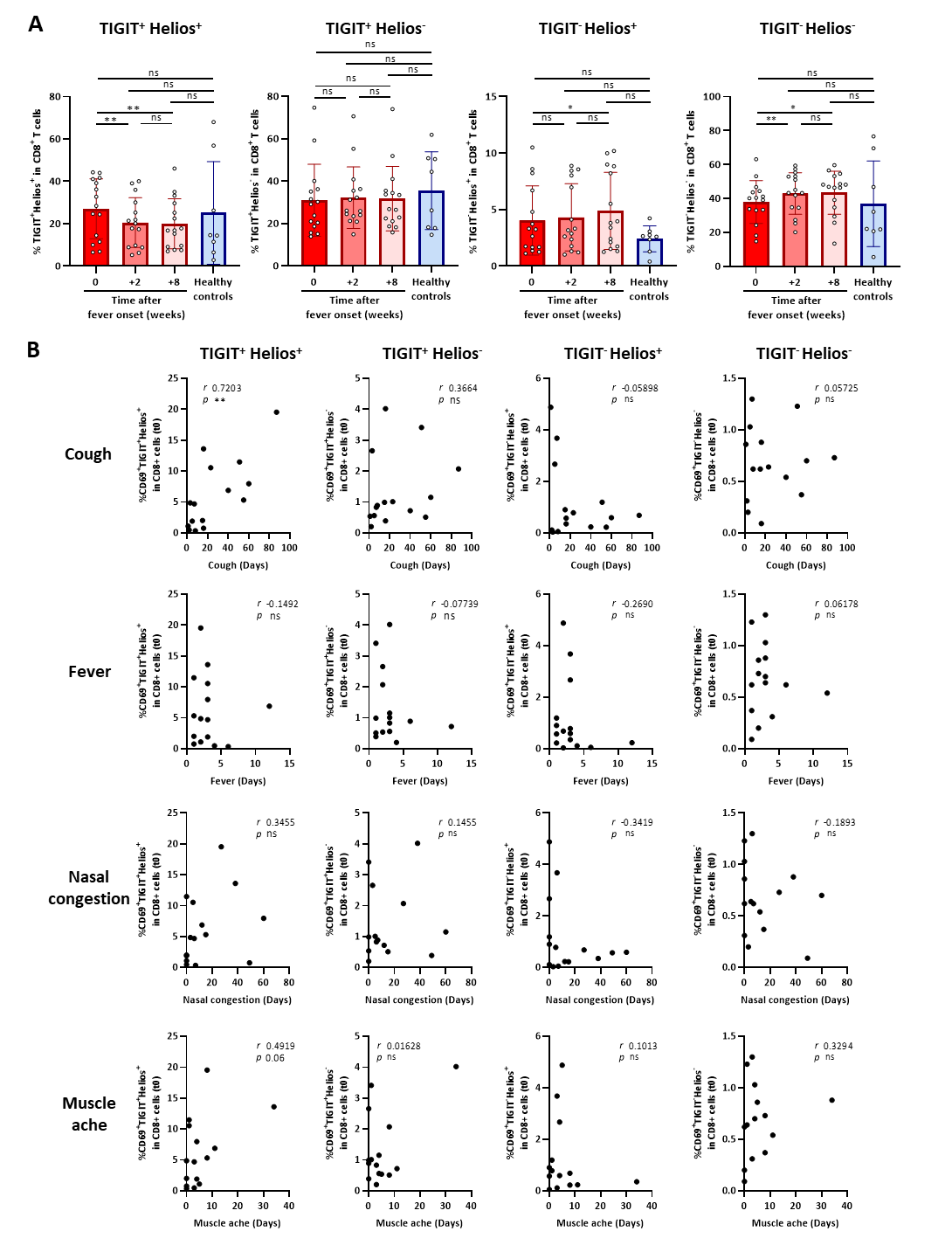


**Supplementary Figure 2. Proportions of TIGIT/Helios cell subsets during influenza A infection and the relationship of their activation to influenza A-associated symptoms.**

(**A**) Bar graphs show the frequency of TIGIT/Helios cell subsets within the total CD8^+^ T-cell population as detected in blood of older adults at the acute phase of influenza (0), and two (+2) and eight (+8) weeks after onset of fever (63-83 years of age, n=15; H3N2 n=12, H1N1 n=3), as well as in older adult asymptomatic controls (61-82 years of age, n=8). (**B**) Relationship between the frequency of CD69^+^ TIGIT/Helios cell subsets within the total CD8^+^ T-cell population and duration of cough, fever, nasal congestion, and muscle ache. Correlations (*r* and *p* values) were assessed by Spearman test. Statistical significance of data presented in the bar graphs (means ± s.d.) was determined using row-matched one-way ANOVA (with Geisser-Greenhouse correction and Dunnett’s post-test) for the difference between the time points and Mann-Whitney *U* test was used to determine the difference between infected and asymptomatic individuals. (**p* <0.05, ***p* <0.01, ns=not significant).


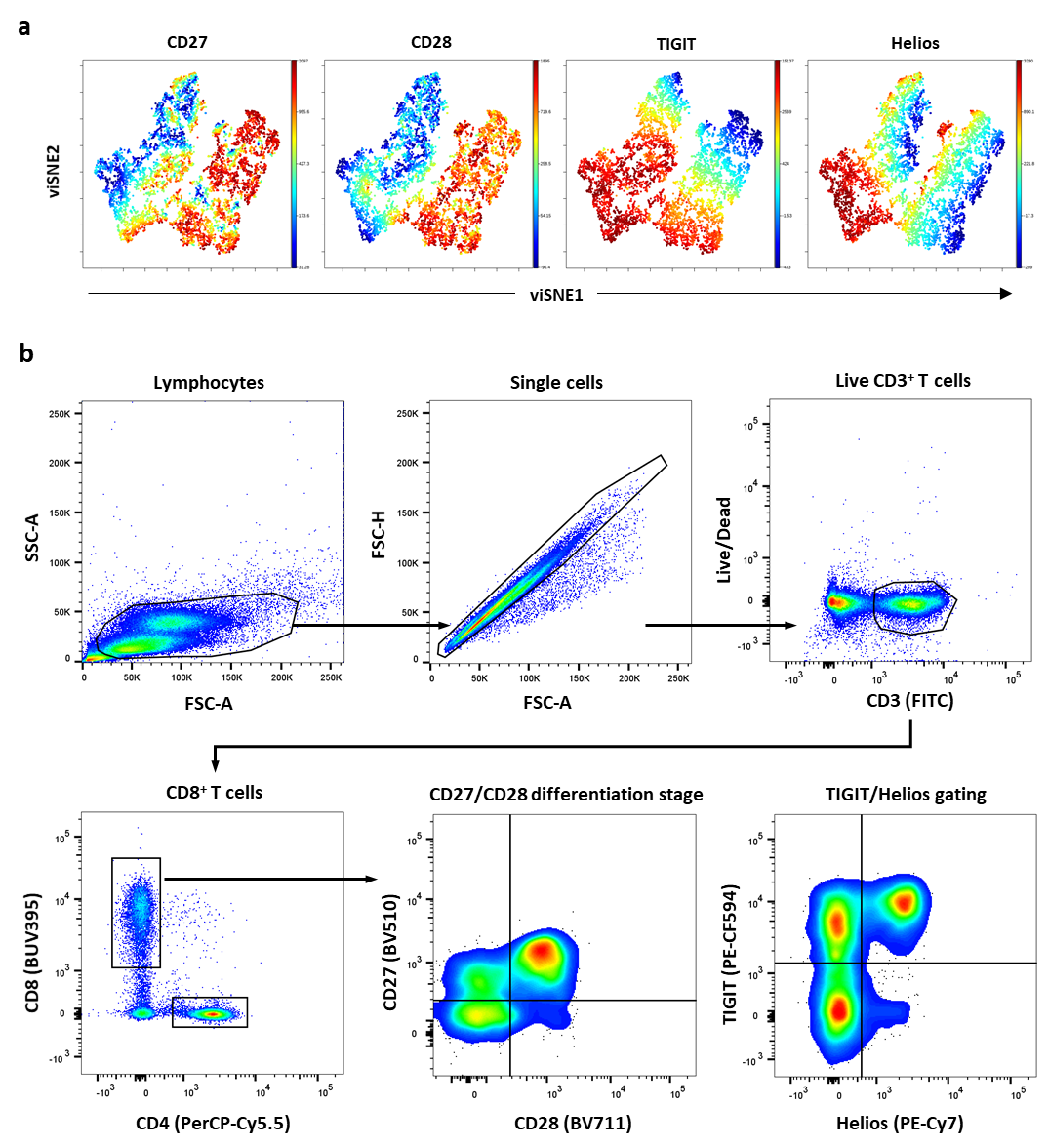


**Supplementary Figure 3. Gating strategy towards CD8^+^ T cells and their separation into TIGIT/Helios subsets and CD27/CD28 subsets.**

(**A**) Fingerprint color dot plots generated by viSNE indicate expression of each of the indicated markers within the CD8^+^ T-cell population of a representative donor as measured by flow cytometry. (**B**) Gating strategy towards stratification of CD8^+^ T cells into TIGIT/Helios cell or CD27/CD28 subsets as performed in healthy individuals using flow cytometry. CD8^+^ T cells identified by gating of total lymphocytes, single cells, and live CD3^+^ T cells. Additionally, CD8^+^ T cells were gated into early (CD27^+^CD28^+^), intermediate (CD27^+^CD28^-^), and late-differentiated (CD27^-^CD28^-^) cells based on their expression of CD27 and CD28. Total CD8^+^ T cells as well as CD8^+^ T cells in one of the three differentiation stages were also separated into TIGIT/Helios cell subsets.
